# Characteristics of the vaginal microbiome in women with premature ovarian insufficiency

**DOI:** 10.1186/s13048-021-00923-9

**Published:** 2021-12-08

**Authors:** Jiaman Wu, Yan Ning, Liya Tan, Yan Chen, Xingxian Huang, Yuanyuan Zhuo

**Affiliations:** 1 Department of Chinese Medicine, Affiliated Shenzhen Maternity and Child Healthcare Hospital, Southern Medical University, Shenzhen, 518028 China; 2 Guangzhou University of Chinese Medicine, Guangzhou, 510006 China; 3 Department of Acupuncture and Moxibustion, Shenzhen Traditional Chinese Medicine Hospital, Shenzhen, 518033 China

**Keywords:** Premature ovarian insufficiency, Vaginal microbiota, 16S rRNA sequencing, Gonadal steroid hormones

## Abstract

**Purpose:**

To investigate the relationship between vaginal microbial community structure and premature ovarian insufficiency (POI).

**Methods:**

Twenty-eight women with POI and 12 healthy women were recruited at Shenzhen Maternity and Child Healthcare Hospital between August and September 2020. Blood samples were collected for glucose tests and detection of sex hormone levels and vaginal secretions were collected for microbial group determination. Vaginal microbial community profiles were analysed by 16S rRNA gene sequencing using the Illumina MiSeq system (Illumina Inc., San Diego, CA, USA).

**Results:**

Compared to the controls, the serum levels of follicle-stimulating hormone, luteinizing hormone, testosterone, and the follicle-stimulating hormone/luteinizing hormone ratio, significantly increased, and oestradiol and anti-Müllerian hormone levels significantly decreased in women with POI. Higher weighted UniFrac values were observed in women with POI than in healthy women. Bacteria in the genera *Lactobacillus*, *Brevundimonas*, and *Odoribacter* were more abundant in the microbiomes of healthy women, while the quantity of bacteria in the genus *Streptococcus* was significantly increased in the microbiomes of women with POI. Moreover, these differences in microbes in women with POI were closely related to follicle-stimulating hormone, luteinizing hormone, oestradiol, and anti-Müllerian hormone levels and to the follicle-stimulating hormone/luteinizing hormone ratio.

**Conclusions:**

Women with POI had altered vaginal microbial profiles compared to healthy controls. The alterations in their microbiomes were associated with serum hormone levels. These results will improve our understanding of the vaginal microbial community structure in women with POI.

**Trial registration:**

CHICTR, ChiCTR2000029576. Registered 3 August 2020 - Retrospectively registered, https://www.chictr.org.cn/showproj.aspx?proj=48844.

## Background

Premature ovarian insufficiency (POI) is an ovarian insufficiency syndrome that occurs in women aged < 40 years and affects 1–2% of women. Recently, POI has shown an increasing incidence [1, 2]. Clinically, the condition is characterised by a continuous decline in ovarian function, resulting in earlier cessation of menstruation than normal [2]. Women with POI can also experience comorbidities, including a low chance of natural conception [3], urogenital atrophy [4], decreased bone mineral density [5], increased risk of autoimmune and thyroid disease [6], cognitive dysfunction [7], shortened life expectancy [8], and cardiovascular disease [9].

The cause of POI is currently unknown; however, a number of potential triggers are associated with the development of the disease, including genetic defects, autoimmune dysfunction, enzyme deficiency, surgical intervention, chemotherapy, radiotherapy, and environmental factors [7, 10]. Several studies have indicated that bacterial vaginosis is associated with infertility [11]. Bacterial vaginosis has also been shown to alter the vaginal microbiome [12]. A previous study demonstrated that the vaginal microbiome plays an important role in the pathophysiology of primary ovarian failure, and the relative abundance of bacteria in the genus *Lactobacillus* was significantly lower in women with primary ovarian failure than in healthy controls [13]. Thus, a relationship may exist between the vaginal microbiome and POI.

In this study, 28 women with POI and 12 healthy women were recruited to study the community profile of the vaginal microbiome. Sequencing of the V3–V4 regions of the 16S rRNA gene in vaginal samples was performed to reveal the differences in the vaginal microbiota between the women with POI and the controls.

## Methods

### Study cohort

Forty women aged 24 to 40 years were recruited at Shenzhen Maternity and Child Healthcare Hospital between August and September 2020. Twenty-eight women with spontaneous POI and 12 healthy women were included in the study. Spontaneous POI was assessed according to criteria described in a previous report [14]. POI was diagnosed if the patient had primary or secondary amenorrhoea for at least 4 months before the age of 40 years and at least two instances of serum follicle-stimulating hormone (FSH) levels exceeding 40 IU/L with an interval of 4–6 weeks. All control women had normal ovarian function, without a history of menstrual dysfunction and infertility and with regular menstruation and normal FSH levels (< 10 IU/L). Participants were excluded if they had the following conditions: non-46-XX karyotype, family history of POI, pregnancy, tumour, chronic diarrhoea, autoimmune diseases, use of antibiotics/medications within the preceding 3 months, pelvic surgery, gastrointestinal disease, active infections, body mass index (BMI) of < 18.5 or > 23.9 kg/m^2^, smoking, or were undergoing chemotherapy or radiotherapy treatment. Clinical characteristics were obtained from the participants’ health records.

The study protocol was approved by the ethics committee of Shenzhen Maternity and Child Healthcare Hospital (Approval number: SFYLS2020–005). Written informed consent was obtained from all participants prior to enrolment. This study was registered at Chinese Clinical Trial Registry (registration No. ChiCTR2000029576).

### Sampling

Blood samples were collected for the glucose (GLU) test and sex hormone levels detection on the third day of menstruation. Patients with amenorrhoea provided blood samples whenever they were available. The serum hormone levels were tested using enzyme-linked immunosorbent assay (ELISA) kit (Sino-UK Institute of Biological Technology, Beijing, China) according to the manufacturer’s instructions in the clinical laboratory. Vaginal secretions from the vaginal posterior fornix were collected using empty sterile collection tubes with an inbuilt sterile swab. Samples were stored at − 80 °C until further analysis.

### DNA extraction and sequencing

DNA was extracted from vaginal samples. DNA quality was assessed using a NanoDrop™ 2000 ultraviolet spectrophotometer (Thermo Fisher Scientific, Waltham, MA, USA) and electrophoresis on a 1% agarose gel. The 16S rRNA gene was amplified using the 338F forward primer 5′-ACTCCTACGGGAGGCAGCAG-3′ and 806R reverse primer 5′-GGACTACHVGGGTWTCTAAT-3′, targeting the variable (V3–V4) region. All samples were pooled and sequenced using the Illumina MiSeq system (Illumina Inc., San Diego, CA, USA). Raw sequencing data were deposited into the NCBI Sequence Read Archive database (SRA BioProject ID: PRJNA738630).

### Sequencing data analysis

The custom Perl script was used to split the sequencing reads from each sample according to the dual index. QIIME 2 (version 2020.08) was used to process the sequence reads [15]. First, we used the command ‘qiime tool import’ to import the sequence data into a QIIME 2 artefact. Second, we used the command ‘qiime data2 denoise-paired’ to exclude chimeric sequences and phiX sequences from the sequence reads. Third, the command ‘qiime2 feature-classifier classify-sklearn’ was used against the Greengenes (13_8 revision) database to assign the taxonomy. The Shannon index was used to represent alpha diversity, which considers both the richness and evenness of microbial communities. The weighted UniFrac distance was used to identify the differences between samples. Both the Shannon index and the UniFrac distance were generated with the command ‘qiime phylogeny align-to-tree-mafft-fasttree’ and ‘qiime diversity core-metrics-phylogenetic’ at a sample depth of 1000. Principal coordinate analysis was then performed based on the weighted UniFrac distance. PICRUSt2.0 (Phylogenetic Investigation of Communities by Reconstruction of Unobserved States) [16] was used to predict functional composition and abundance.

### Statistical analysis

Data analysis was performed using R software (R Foundation for Statistical Computing, Vienna, Austria). Unpaired *t*-tests were used for comparisons of normally distributed data sets and Wilcoxon rank-sum tests were used for non-normally distributed data. Normally distributed data are expressed as means ± standard deviations and non-normally distributed data are expressed as numbers (percentages). Correlation analysis was performed using Pearson’s correlation coefficient. Statistical significance was set at *p* < 0.05.

Linear discriminant analysis (LDA) effect size analysis was used to determine the significant differences in microbes between the POI and control groups with an LDA cut-off score of 2.0 [17]. The System-Theoretic Accident Model and Processes (STAMP) software [18] was used to investigate the functional differences between the POI and control groups. A ‘support vector machine’ algorithm with 5-fold cross-validation was used to build a classification model to identify women with POI based on microbial community. Receiver operating characteristic curves were generated, and the area under the curve was calculated to evaluate the model’s performance.

## Results

### Participant characteristics

Twenty-eight women with POI and 12 healthy controls were recruited for analysis. The clinical characteristics of the two groups are shown in Table 1. The mean ages in the POI and control groups were 34.61 ± 4.37 and 32.5 ± 3.87 years, respectively. The mean BMIs in the POI and control groups were 21.01 ± 1.19 and 20.81 ± 1.60, respectively. Age and BMI did not significantly differ between the two groups. Progesterone (P), prolactin (PRL), and GLU levels were not significantly different between women with and without POI. Women with POI had significantly higher levels of FSH, luteinizing hormone (LH), and testosterone and a higher FSH/LH ratio, but significantly lower oestradiol (E2) and anti-Müllerian hormone (AMH) levels compared to the control women.

**Table 1:** Demographic and clinical characteristics of the two groups

	POI group (***n*** = 28)^**a**^	Control group (***n*** = 12)^**a**^	***P***-value
Age (years)	34.61 ± 4.37	32.5 ± 3.87	0.16
BMI (kg/m^2^)	21.01 ± 1.19	20.81 ± 1.60	0.66
FSH (mIU/mL)	45.54 ± 30.68	5.41 ± 1.87	< 0.01**
LH (mIU/mL)	16.15 ± 8.35	4.05 ± 1.23	< 0.01**
E2 (pg/L)	32.61 ± 18.86	55.08 ± 9.87	< 0.01**
P (nmol/L)	0.52 ± 0.37	0.35 ± 0.17	0.14
T (nmol/L)	0.44 ± 0.20	0.29 ± 0.12	0.02*
PRL (nmol/L)	14.74 ± 8.51	11.81 ± 4.31	0.27
AMH (ng/mL)	0.54 ± 0.36	4.34 ± 2.15	< 0.01**
FSH/LH (ratio)	2.74 ± 0.71	1.33 ± 0.18	< 0.01**
GLU (nmol/L)	5.18 ± 0.45	4.92 ± 0.28	0.08

*POI* Premature ovarian insufficiency, *BMI* Body mass index, *FSH* Follicle-stimulating hormone, *LH* Luteinizing hormone, *E2* Oestradiol, *P* Progesterone, *T* Testosterone, *PRL* Prolactin, *AMH* Anti-Müllerian hormone, *GLU* Glucose

*When *P* < 0.05, “*” marks the significant

**When *P* < 0.01, “**” marks significance

^a^Normally distributed data are expressed as means ± standard deviations

### Overall community structure of the vaginal microbiome of women with POI

To evaluate the community structure of the vaginal microbiota in women with and without POI, sequencing was performed on the V3–V4 regions of the 16S rRNA. In total, 1,903,682 usable reads (47,592 ± 9664 reads per sample) were obtained from all 40 samples, and the mean and median sequence lengths were 423 and 428 base pairs, respectively. The number of reads analysed did not differ between the POI and control samples (47,805 ± 10,475 vs 47,093 ± 7834; *p* = 0.83), indicating comparable and adequate sequencing coverage.

**Fig. 1:**
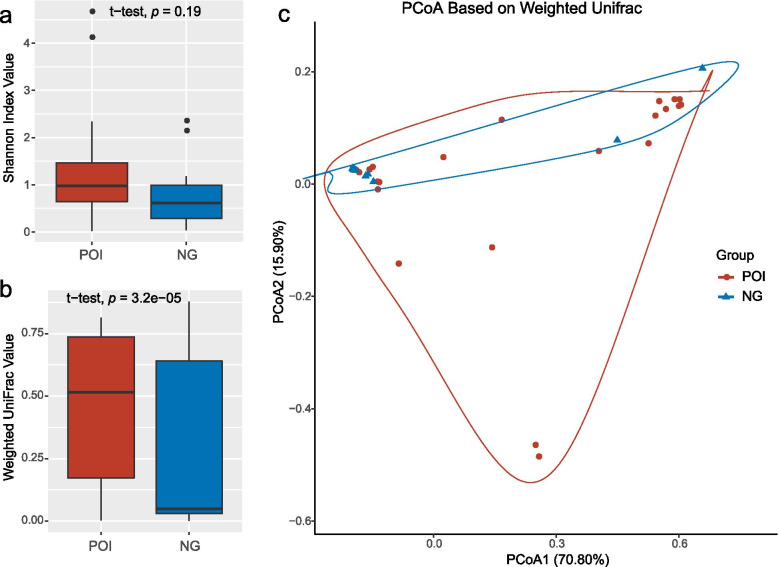
Overall structural differentiation of the vaginal microbiota between the POI and control groups. **a** Shannon index between the two groups. **b** Weighted UniFrac value between the two groups. **c** PCoA plot based on weighted UniFrac value. NG, healthy control group; PCoA, principal coordinates analysis; POI, premature ovarian insufficiency

To explore the dissimilarity in the vaginal microbiota between the two groups, the alpha and beta diversities were represented using the Shannon and weighted UniFrac distance indices, respectively. The POI group exhibited a higher mean Shannon index than the control group; however, this was not statistically significant (1.21 ± 1.08 vs 0.80 ± 0.76; *p* = 0.19; Fig. 1a). The average weighted UniFrac value was significantly higher in the POI group than in the control group (0.45 ± 0.29 vs 0.26 ± 0.34; *p* < 0.01; Fig. 1b). Further, the results of principle coordinates analysis performed based on the weighted UniFrac distance showed that the microbial communities were not compositionally different between the two groups (Fig. 1c).

### Characterising the vaginal microbiome in the POI group

**Fig. 2:**
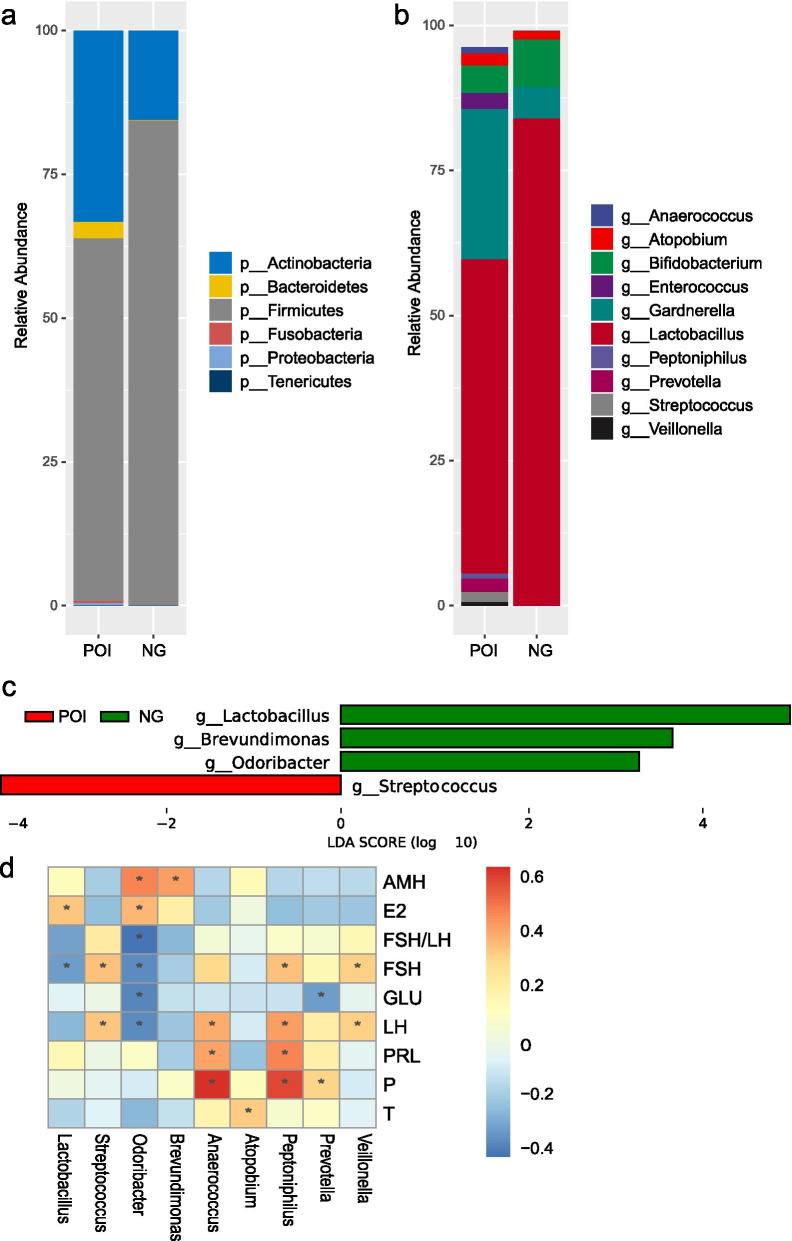
Microbial community profiles of the vaginal microbiota in the POI and control groups. **a** Relative abundances of the dominant phylum. **b** Relative abundances of the top 10 genera. **c** Microbes that significantly differed between the POI and control groups. **d** Pearson correlation between microbes and serum hormones. AMH, anti-Müllerian hormone; E2, oestradiol; FSH, follicle-stimulating hormone; GLU, glucose; LDA, linear discriminant analysis; LH, luteinizing hormone; NG, healthy control group; P, progesterone; POI, premature ovarian insufficiency; PRL prolactin; T, testosterone

Vaginal microbiome communities were dominated by the phyla Actinobacteria, Bacteroidetes, Firmicutes, Fusobacteria, Proteobacteria, and Tenericutes in both the POI and control groups (Fig. 2a). Firmicutes were predominant, accounting for 63.08% ± 40.94 and 84.17% ± 35.40% of the total microbiota on average in the POI and control groups, respectively. The top 10 most abundant genera in both groups were *Anaerococcus*, *Atopobium*, *Bifidobacterium*, *Enterococcus*, *Gardnerella*, *Lactobacillus*, *Peptoniphilus*, *Prevotella*, *Streptococcus*, and *Veillonella* (Fig. 2b). Compared to the control group, the POI group had increased quantities of *Anaerococcus* (1.05% vs 0.01%), *Atopobium* (2.04% vs 1.47%), *Enterococcus* (2.69% vs 0.00%), *Gardnerella* (25.94% vs 5.51%), *Peptoniphilus* (0.84% vs 0.01%), *Prevotella* (2.32% vs 0.07%), *Streptococcus* (1.76% vs 0.16%), and *Veillonella* (0.65% vs 0.01%), but decreased quantities of *Bifidobacterium* (4.80% vs 8.16%) and *Lactobacillus* (54.18% vs 83.94%).

The significance of the different genera in the POI and control groups was investigated using LDA effect size analysis. Significantly lower abundances of the genera *Lactobacillus* (LDA = 5.16, *p* = 0.02), *Odoribacter* (LDA = 3.42, *p* < 0.01), and *Brevundimonas* (LDA = 3.81, *p* = 0.0) and a significantly higher abundance of *Streptococcus* (LDA = 3.90, *p* = 0.04) were observed in the POI group compared to the control group (Fig. 2c).

Pearson correlation analysis was performed to evaluate the association between the different microbes and serum hormones. The relative proportion of the genus *Lactobacillus* was significantly correlated with E2 (*R* = 0.34, *p* = 0.03) and FSH (*R* = − 0.31, *p* = 0.049) levels. The genus *Brevundimonas* was significantly positively correlated with AMH levels (*R* = 0.43, *p* = 0.006). The genus *Odoribacter* was significantly positively correlated with AMH (*R* = 0.48, *p* = 0.002) and E2 (R = 0.38, *p* = 0.015) levels, and negatively correlated with the FSH/LH ratio (*R* = − 0.42, *p* = 0.008), FSH levels (*R* = − 0.36, *p* = 0.024), GLU levels (*R* = − 0.37, *p* = 0.021), and LH levels (*R* = − 0.35, *p* = 0.025). The genus *Streptococcus* was significantly positively correlated with FSH (*R* = 0.35, *p* = 0.025) and LH (*R* = 0.35, *p* = 0.029) levels (Fig. 2d).

Correlations between the abundant genera and serum hormones were also investigated. The abundance of the genus *Anaerococcus* was positively correlated with levels of LH (*R* = 0.40, *p* = 0.011), PRL (*R* = 0.42, *p* = 0.007), and P (*R* = 0.65, *p* = 0.0001). The genus *Atopobium* was positively correlated with P levels (*R* = 0.33, *p* = 0.035). The genus *Peptoniphilus* was positively correlated with levels of FSH (*R* = 0.35, *p* = 0.026), LH (*R* = 0.43, *p* = 0.005), PRL (*R* = 0.49, *p* = 0.001), and P (*R* = 0.61, *p* = 0.001). The genus *Prevotella* was negatively correlated with GLU levels (*R* = − 0.31, *p* = 0.048), but positively correlated with P levels (*R* = 0.31, *p* = 0.049). The genus *Veillonella* was significantly positively correlated with FSH (*R* = 0.33, *p* = 0.039) and LH (*R* = 0.33, *p* = 0.038) levels (Fig. 2d).

A classification model was then constructed based on differentially expressed and abundant genera using the support vector machine algorithm with 5-fold cross-validation. The area under the curve was 0.79, demonstrating that POI could be accurately predicted based on microbial community.

### Different metabolic functions between the POI and control groups

**Fig. 3:**
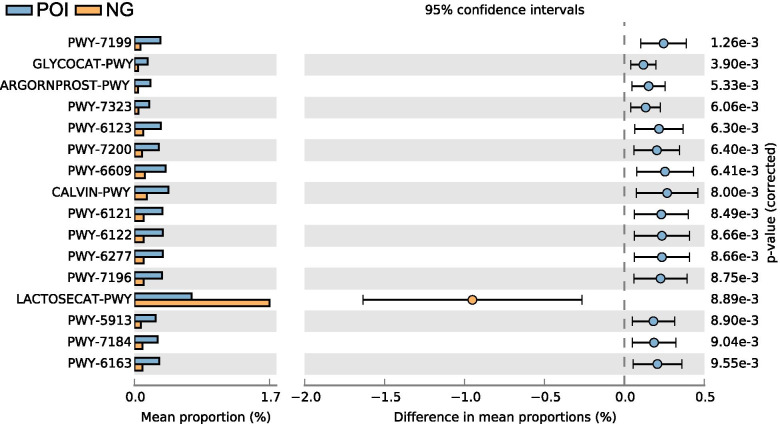
Metabolic pathways that differed significantly between the POI and control groups. NG, healthy control group; POI, premature ovarian insufficiency; PWY-7199, pyrimidine deoxyribonucleosides salvage; GLYCOCAT-PWY, glycogen degradation I; ARGORNPROST-PWY, L-arginine degradation; PWY-7323, superpathway of GDP-mannose-derived O-antigen building blocks biosynthesis; PWY-6123, inosine-5′-phosphate biosynthesis I; PWY-7200, superpathway of pyrimidine deoxyribonucleoside salvage; PWY-6609, adenine and adenosine salvage III; CALVIN-PWY, Calvin-Benson-Bassham cycle; PWY-6121, 5-aminoimidazole ribonucleotide biosynthesis I; PWY-6122, 5-aminoimidazole ribonucleotide biosynthesis II; PWY-6277, superpathway of 5-aminoimidazole ribonucleotide biosynthesis; PWY-7196, superpathway of pyrimidine ribonucleosides salvage; LACTOSECAT-PWY, lactose and galactose degradation I; PWY-5913, (partial TCA cycle obligate autotrophs); PWY-7184, pyrimidine deoxyribonucleotides de novo biosynthesis I; PWY-6163, chorismate biosynthesis from 3-dehydroquinate

Using PICRUSt2.0, the metabolic pathways of the two groups based on the Metacyc database were performed. STAMP analysis identified 16 significantly different pathways between the two groups (Fig. 3), including PWY-7199 (pyrimidine deoxyribonucleosides salvage), GLYCOCAT-PWY (glycogen degradation I), ARGORNPROST-PWY (L-arginine degradation), PWY-7323 (superpathway of GDP-mannose-derived O-antigen building blocks biosynthesis), PWY-6123 (inosine-5′-phosphate biosynthesis I), PWY-7200 (superpathway of pyrimidine deoxyribonucleoside salvage), PWY-6609 (adenine and adenosine salvage III), CALVIN-PWY (Calvin-Benson-Bassham cycle), PWY-6121 (5-aminoimidazole ribonucleotide biosynthesis I), PWY-6122 (5-aminoimidazole ribonucleotide biosynthesis II), PWY-6277 (superpathway of 5-aminoimidazole ribonucleotide biosynthesis), PWY-7196 (superpathway of pyrimidine ribonucleosides salvage), LACTOSECAT-PWY (lactose and galactose degradation I), PWY-5913 (partial TCA cycle [obligate autotrophs]), PWY-7184 (pyrimidine deoxyribonucleotides de novo biosynthesis I), and PWY-6163 (chorismate biosynthesis from 3-dehydroquinate). Excluding the LACTOSECAT-PWY, the remaining 15 pathways were significantly enriched in women with POI.

## Discussion

Previous reports indicate that the vaginal microbiome contributes significantly to female and neonatal health [19, 20]. In this study, we aimed to determine the overall composition of the vaginal microbiota in women with POI. The vaginal microbiota of both the POI and control groups were composed primarily of the phyla Actinobacteria, Bacteroidetes, and Firmicutes. The genus *Lactobacillus* was predominant, with low proportions of the genera *Anaerococcus*, *Atopobium*, *Bifidobacterium*, *Enterococcus*, *Gardnerella*, *Peptoniphilus*, *Prevotella*, *Streptococcus*, and *Veillonella*. These results are in accordance with previous observations [20, 21]. Increased patterns of alpha and beta diversity were observed in the POI group, which is consistent with the findings in women with primary ovarian failure [13].

Previous studies have shown that inflammatory and autoimmune responses are closely related to ovarian function and POI [6, 22]. In this study, compared to the control group, the genera *Lactobacillus*, *Odoribacter*, and *Brevundimonas* were significantly decreased and the genus *Streptococcus* significantly increased in the POI group. Lactobacilli can promote interleukin (IL)-22 secretion and prevent autoimmune diseases by stimulating the production of antimicrobial peptide [23] and can reinforce the mononuclear phagocytic response [24]. Some species of *Odoribacter* can induce Th17 cells and protective immunity by promoting IL-1β and IL-6 signalling [25]. Some *Streptococcus* species produce toxins that activate innate and adaptive host immune responses [26]. *Streptococcus agalactiae* (also known as group B *Streptococcus*) is a common bacterial infection during pregnancy, preterm birth, and neonatal infection. Specific *Lactobacillus* strains could serve as probiotics to prevent vaginal group B *Streptococcus* colonisation [27]. Moreover, *Gardnerella vaginalis* can activate NF-κB to promote tumour necrosis factor α secretion [28]; the inflammatory response induced by *G. vaginalis* can be inhibited by *Lactobacillus* [13]. Increases in *Streptococcus*, *Gardnerella* and a decrease in *Lactobacillus* species were observed in women with POI. The changes induced by the differing vaginal microbiome in the POI group might affect autoimmunity, which could be related to the development of POI.

Sex hormone levels can affect the defensive ability of the female genital tract and the resident vaginal microbiome during the reproductive years [29, 30]. Thus, changes in sex hormones play an important role in the vaginal microbiome [31]. Oestrogen promotes hyperplasia and increased glycogen production [32]; glycogen can then be converted into lactic acid by lactobacilli, the dominant bacteria in the vagina. This helps maintain the acidic environment of the vagina, inhibiting the growth of pathogens, and strengthening the immune system [33, 34]. In this study, the proportion of *Lactobacillus* was positively correlated with oestrogen levels, but negatively correlated with FSH levels. Significant decreases in *Lactobacillus* abundance and oestrogen levels and a significant increase in FSH levels were observed in women with POI. These findings are consistent with the characteristics of women in the initial stages of menopause, who have reduced oestrogen levels, increased FSH levels, and colonisation of a larger number of mixed bacteria due to the change in vaginal pH from acidic to weakly acidic [35, 36]. Moreover, AMH, FSH, LH, PRL, P, and testosterone levels were also related to some microbes, including *Streptococcus*, *Odoribacter*, *Brevundimonas*, *Anaerococcus*, *Atopobium*, *Peptoniphilus*, *Prevotella*, and *Veillonella*, increasing the evidence that an altered vaginal microbiota is associated with sex hormones. Accumulating evidence indicates that oestrogen can regulate POI-related symptoms, including GLU and lipid metabolism, bone formation, and inflammatory responses [37, 38], and GLU level was negatively correlated with genera *Odoribacter* and *Prevotella* in this study. In addition, PRL can inhibit FSH and gonadotropin-releasing hormone to promote fertility [39], which was positively correlated with *Anaerococcus* and *Peptoniphilus*. However, due to the limited data in this study, the mechanism underlying the relationship between these microbes and sex hormones cannot be fully clarified.

Ovarian function is known to be susceptible to damage due to galactose and galactose metabolite accumulation [40]. A previous study found that galactose suppressed the number of ovarian follicles and steroid secretion [41]. Galactose metabolites, including galactose-1-phosphate, galactitol, and uridine diphosphate galactose, play important roles in interfering with ovarian apoptosis and gonadotrophin signalling [40]. The functional analysis results showed that the activity of the LACTOSECAT-PWY pathway decreased significantly in women with POI compared to the control group. This demonstrated that galactose may accumulate in women with POI, damaging ovarian function. The ARGORNPROST-PWY pathway was significantly enriched in women with POI. L-arginine is associated with regulation of ovarian function [42, 43]. This may be unfavourable for ovarian function. In addition, DNA damage results in reproductive dysfunction by activating the mitochondrial apoptosis pathway [44]. Our results showed that many pathways related to ribonucleotide biosynthesis were altered in women with POI. Thus, dysbiosis of the vaginal microbiome in women with POI is closely related to ovarian function.

There are some limitations of this study. The sample size was small, the recruited participants were all from the same hospital, and we could only determine association, not causality. Thus, future studies should include a larger sample size and samples from multiple centres. Metagenome sequencing to study the microbiome at lower level, and transplantation of vaginal microbiome from women with POI to germ-free mice should be considered to investigate the potential causal mechanism.

In summary, this study demonstrated the dysbiosis of the vaginal microbiome in women with POI and confirmed that *Lactobacillus* is the predominant genus in the vaginal microbial community of women with POI. The changes in microbial communities are closely related to serum hormones, and the metabolic function of the microbes affects ovarian function. Our results lay a foundation for revealing the interaction between the vaginal microbiota and POI.

## Data Availability

The dataset supporting the conclusions of this article is available in the NCBI Sequence Read Archive database (SRA BioProject ID: PRJNA738630).
